# Generalized Discriminant Orthogonal Nonnegative Tensor Factorization for Facial Expression Recognition

**DOI:** 10.1155/2014/608158

**Published:** 2014-03-26

**Authors:** Zhang XiuJun, Liu Chang

**Affiliations:** ^1^College of Information Science and Technology, Chengdu University, Chengdu 610106, China; ^2^Key Laboratory of Pattern Recognition and Intelligent Information Processing in Sichuan, Chengdu 610106, China

## Abstract

In order to overcome the limitation of traditional nonnegative factorization algorithms, the paper presents a generalized discriminant orthogonal non-negative tensor factorization algorithm. At first, the algorithm takes the orthogonal constraint into account to ensure the nonnegativity of the low-dimensional features. Furthermore, the discriminant constraint is imposed on low-dimensional weights to strengthen the discriminant capability of the low-dimensional features. The experiments on facial expression recognition have demonstrated that the algorithm is superior to other non-negative factorization algorithms.

## 1. Introduction

Over the past few years, the nonnegative matrix factorization algorithm (NMF) [1] and its variants have proven to be useful for several problems, especially in facial image characterization and representation problems [2–8]. The idea of nonnegative factorization is partly motivated by the biological fact that the firing rates in visual perception neurons are nonnegative.

However, NMF and its variants have some drawbacks. First of all, NMF requires that all object images should be vectorized in order to find the non-negative decomposition. This vectorization leads to information loss, since the local structure of the image is lost. Moreover, NMF is not unique [9, 10]. In order to remedy these drawbacks, non-negative tensor factorization (NTF) has been proposed [11–13]. NTF represents a facial expression database as a three-order tensor. The tensor representation avoids the vectorization operation and preserves the structure of the data. Under some mild conditions, NTF is unique. Existing NMF and NTF algorithms project data into low-dimensional space with the inverse or pseudoinverse of the basis images, so both of them cannot guarantee the nonnegativity of low-dimensional features, which restricts the application of non-negative factorization in real world. Furthermore, NTF do not take into account class information in data samples. Actually, it is believed that those features with discriminant constraints are of great importance for pattern recognition. Reference [14] develops a discriminant non-negative tensor factorization algorithm (DNTF), which adds fisher discriminant constraint into the objective function. But like other discriminant non-negative matrix factorizations [6, 15–18], DNTF employed discriminant analysis on the representation coefficients and not on the actual features used in the recognition procedure. The actual features used for recognition are derived from the projection of data samples to the bases matrix and only implicitly depend on the representation coefficients.

Based on the above analysis, the paper proposes a generalized discriminant orthogonal non-negative tensor factorization algorithm (GDONTF), which makes full use of the class information and imposes the orthogonal constraint to the objective function. The algorithm not only guarantees the non-negativity of low-dimensional features, but also generalizes discriminant constraints to low-dimension features. The experiments on facial expression recognition indicate that GDONTF achieves better performance than other non-negative factorization algorithms.

## 2. Generalized Discriminant Orthogonal Non-Negative Tensor Factorization

Consider an *N* order tensor *X* ∈ ℝ^*d*_1_×*d*_2_⋯×*d*_*N*_^, every data sample *X*_*i*_ is an *n* − 1 order tensor; that is, *X*_*i*_ ∈ ℝ^*d*_1_×*d*_2_⋯×*d*_*n*−1_^, in which *d*_1_, *d*_2_ ⋯ *d*_*N*−1_, is the dimensionality and *d*_*N*_ is the number of data set. The data set is divided into *C* classes. Data samples belonging to class *c* denote *V*(*c*); the number of data samples in *V*(*c*) is *N*_*c*_. In order to guarantee the non-negativity of low-dimensional features and take use of the class information, we propose generalized discriminant orthogonal non-negative tensor factorization algorithm; the objective function of which is defined as follow:

(1) O=||X−∑r=1RU:,r(1)∘  U:,r(2)⋯∘U:,r(N)||2+αtr[Sw]−βtr[Sb]s.t. U(n)U(n)T=I n=1,2,…,N−1.

In which, *α* ≥ 0, *β* ≥ 0, *U*_*ij*_^(*n*)^ ≥ 0, *n* = 1,2,…, *N*, *I* is the identity matrix and *S*_*w*_ and *S*_*b*_ are the within- and between-class scatter matrices of the low-dimensional features, respectively. Because *U* ^(*n*)^ *U* ^(*n*)^ ^*T*^ = *I*, low-dimensional features can be computed as follows:

(2) $$\begin{array}{c} h_{i}={\left(U^{\left(N-1\right)}⊙U^{\left(N-2\right)}⊙\cdots ⊙U^{\left(2\right)}⊙U^{\left(1\right)}\right)}^{T}\bar{X_{i}}=W^{T}\bar{X_{i}}, \end{array}$$

where the basis matrix *W* = *U* ^(*N*−1)^⊙*U* ^(*N*−2)^⊙⋯⊙*U* ^(2)^⊙*U* ^(1)^. Let *h*_*i*_ be the low-dimensional features of the sample *X*_*i*_; then the feature matrix *H* ∈ ℝ^*d*×*M*^ consists of all low-dimensional features, *d* is the low dimensionality of samples, and *M* is the number of all samples. Actually, the separability of the weight coefficient has nothing to do with the recognition accuracy, while the class separability of the low-dimensional features has a great influence on the recognition accuracy. Consequently, the within- and between-class scatter matrices are defined as follows:

(3) Sw=∑c=1C ∑ui∈V(c)(hi−mc)(hi−mc)T=∑c=1C ∑ui∈V(c)(WTXi¯−mc)(WTXi¯−mc)T,Sb=∑c=1CNc(mc−m)(mc−m)T,

where *m*_*c*_ is the mean of the low-dimensional features in the class *c* and *m* is the mean of all low-dimensional features. The objective function in (1) can be written as the following optimization problem:

(4) min⁡U(n) ||X−∑r=1RU:,r(1)∘U:,r(2)⋯∘U:,r(N)||2+αtr[Sw]−βtr[Sb]s.t. Uij(n)≥0   U(n)TU(n)=I, n=1,2,…,N−1.

Since the basis matrix *W* consists of the projection matrices *U* ^(*n*)^, *n* = 1,2,…, *N* − 1, we solve the projection matrices *U* ^(*n*)^, *n* = 1,2,…, *N* − 1, and the weight matrix *U* ^(*N*)^, respectively, to deal with the optimization problem (4). First of all, we formulate the Lagrange multipliers out of the constrained optimization problem in (4):

(5) f(U(n),λ)=||X(n)−U(n)GT||2+αtr[Sw]−βtr[Sb]+Tr[λ(U(n)TU(n)−I)],

where *G* = *U* ^(*N*)^⊙⋯⊙*U* ^(*n*+1)^⊙*U* ^(1)^⊙*U* ^(2)^⊙⋯⊙*U* ^(*n*−1)^.

Take the derivative of *f*(*U* ^(*n*)^, *λ*) with respect to *U* ^(*n*)^ and *λ*, *n* = 1,2,…, *N* − 1; we have

(6) ∂f∂U(n)=−2X(n)G+2U(n)GTG+2U(n)λ+α∂Tr(Sw)∂U(n) −β∂Tr(Sb)∂U(n)=−2X(n)G+2U(n)GTG+2U(n)λ+α∇Tr(Sw) −β∇Tr(Sb),

(7) $$\begin{array}{c} \frac{\partial f}{\partial \lambda }={U^{\left(n\right)}}^{T}U^{\left(n\right)}-I. \end{array}$$

Set (6) and (7) to zeros; we get

(8) −2X(n)G+2U(n)GTG+2U(n)λ+α∇Tr(Sw)  −β∇Tr(Sb)=0,

(9) $$\begin{array}{c} {U^{\left(n\right)}}^{T}U^{\left(n\right)}=I. \end{array}$$

Left multiply both side of (8) by *U* ^(*n*)^ ^*T*^; we immediately have

(10) −2U(n)TX(n)G+2U(n)TU(n)GTG+2U(n)TU(n)λ  +αU(n)T∇Tr(Sw)−βU(n)T∇Tr(Sb)=0.

Therefore, the update rule for *U* ^(*n*)^ is

(11) $$\begin{array}{c} U^{{\left(n\right)}^{t+1}}=U^{{\left(n\right)}^{t}}*\frac{2X_{\left(n\right)}G+\beta \nabla Tr\left(S_{b}\right)}{2U^{\left(n\right)}G^{T}G+2U^{\left(n\right)}\lambda +\alpha \nabla Tr\left(S_{w}\right)}. \end{array}$$

The gradient [∇*Tr*(*S*_*b*_)]_*i*,*j*_ = ∂*Tr*[*S*_*w*_]/∂*u*_*ij*_^(*n*)^ is given by

(12) ∂Tr[Sw]∂uij(n)  =∂∑p∑c=1C ∑hp∈V(c)(hp−mc)(hp−mc)T∂uij(n)  =∂∑p∑c=1C ∑hp∈V(c)(WTXp¯−mc)(WTXp¯−mc)T∂uij(n).

Because *W* = (*U* ^(*N*−1)^⊙*U* ^(*N*−2)^⊙⋯⊙*U* ^(2)^⊙*U* ^(1)^), we can get

(13) ∂Tr[Sw]∂uij(n)=(∂∑p∑c=1∑hp∈V(c)((U(N−1)⊙U(N−2)…⊙U(1))T×Xp¯−mc)2)×(∂uij(n))−1=2∑p∑c=1C  ∑hp∈V(c)(WTXp¯−mc)×(∂((U(N−1)⊙U(N−2)…⊙U(1))T×(Xp¯−∑q=1hq∈V(c)NcXq¯)))×(∂uij(n))−1.

Let *U* ^*pn*^ = *U* ^(*N*−1)^⊙*U* ^(*N*−2)^⊙⋯⊙*U* ^(*n*+1)^ and *U* ^*an*^ = *U* ^(*n*−1)^⊙*U* ^(*n*−2)^⊙⋯⊙*U* ^(1)^; we have

(14) ∂Tr[Sw]∂uij(n)=2∑p∑c=1C  ∑hp∈V(c)(WTXp¯−mc) ×(∂((Upn⊙U(n)⊙Uan)T     ×(Xp¯−∑q=1hq∈V(c)NcXq¯)))×(∂uij(n))−1;

Since

(15) Upn⊙U(n)⊙Uan  =[U1pn⊗U1(n)⊗U1an,…,Ujpn⊗Uj(n)⊗Ujan,…]  =[U:,1pn⊗U:,1(n)⊗U:,1an,…,U:,jpn⊗U:,j(n)⊗U:,jan,…]  =[u11pnu11(n)u11an,…,u1jpnu1j(n)u1jan,…,    u1jpnuij(n)u1jan,…,uijpnuij(n)uijan,…].

We have

(16) ∂Tr[Sw]∂uij(n)=2∑p∑c=1C ∑hp∈V(c)(WTXp¯−mc) ×[0,…,0,u1jpnu1jan,…,uijpnuijan,…,0,…,0]Xp¯.

Similarly, we have

(17) ∂Tr[Sb]∂uij(n)=∂∑p∑c=1CNc(mpc−mp)(mpc−mp)T∂uij(n)=∂∑p∑c=1CNc(∑hp∈V(c)NcWTXp−−∑q=1MWTXq¯)2∂uij(n)=2∑p∑c=1CNc(mpc−mp) ×∂(∑hp∈V(c)NcWTXp−−∑q=1MWTXq¯)∂uij(n)=2∑p∑c=1CNc(mpc−mp) ×(∂(U(N−1)⊙U(N−2)⊙⋯⊙U(1))T   ×(∑hp∈V(c)NcXp−−∑q=1MXq−))×(∂uij(n))−1=2∑p∑c=1CNc(mpc−mp) ×[0,…,0,u1jpnu1jan,…,uijpnuijan,…,0,…,0] ×(∑hp∈V(c)NcXp−−∑q=1MXq−).

To solve the weight matrix *U* ^(*N*)^, the objective function is

(18) f(U(N))=||X(N)−U(N)(U(N−1)⊙U(N−2)⊙⋯⊙U(1))T||2.

The gradient functon is

(19) $$\begin{array}{c} g\left(U^{\left(N\right)}\right)=-2X_{\left(N\right)}W+2U^{\left(N\right)}W^{T}W, \end{array}$$

where *W* = *U* ^(*N*−1)^⊙*U* ^(*N*−2)^⊙⋯⊙*U* ^(1)^.

Consequently, the update rules of *U* ^(*N*)^ are

(20) $$\begin{array}{c} U_{ij}^{{\left(N\right)}^{t+1}}=U_{ij}^{{\left(N\right)}^{t}}\frac{{\left(X_{\left(N\right)}W\right)}_{ij}}{{\left(U^{\left(N\right)}W^{T}W\right)}_{ij}}. \end{array}$$

## 3. Experiments

We have conducted facial expression recognition in order to compare the GDONTF with other algorithms such as NMFOS [19], DNMF [6], FisherNMF [16], and DNTF [14]. Because these algorithms calculate low-dimension features in iteration form, the iteration number is 100. For NMFOS and GDONTF, *λ* = 1. *γ* = 0.5 in DNMF and *α* = 1 in FisherNMF. All low-dimension features are classified by SVM with linear kernel.

**Figure 1:**
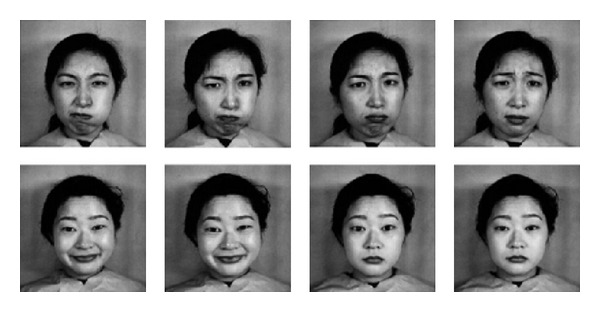
Some images in the Jaff facial expression database.

**Figure 2:**
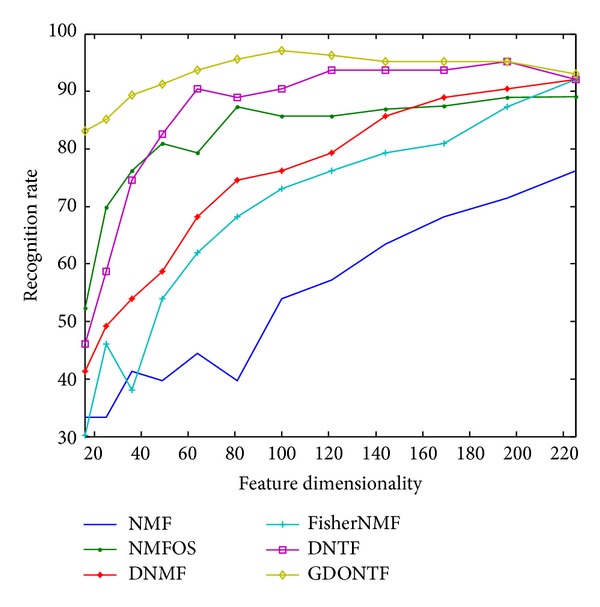
Facial expression recognition rate versus dimensionality in Jaff database.

The database used for the facial expression recognition experiments is Jaff facial expression database [20]. The database contains 213 images of ten Japanese women. Each person has two to four images for each of the seven expressions: neutral, happy, sad, surprise, anger, disgust, and fear. Each image is resized into 32 × 32. A few examples are shown in Figure 1. We randomly select 20 images from each expression for training; the rest is used for testing. The recognition rates with various dimensionalities of different algorithms are shown in Figure 2.  Table 1 shows the best recognition rates of the above algorithms. Because NMF is unsupervised learning algorithm, it has the lowest recognition rates. DNMF and FisherNMF have better recognition rates with supervised learning. It is interesting that NMFOS is superior to DNMF and FisherNMF when the feature dimensionality is from 16 to 160 and is better than DNTF when the feature dimensionality is from 16 to 40, which also illustrates the validity of the orthogonal constraint. It is obvious that GDONTF outperforms other algorithms and the best recognition rate is up to 97.07%.

## 4. Conclusion

In this paper, a generalized discriminant orthogonal non-negative tensor factorization algorithm is proposed considering the orthogonal constraint and the discriminant constraint. For the algorithm, the non-negativity of the low-dimensional features is preserved due to the orthogonal constraint for either training samples or testing samples. In order to enhance the recognition accuracy, the discriminant is conducted on low-dimensional features instead of the weight coefficient of the basis images. The experiments also validate the performance of the algorithm.

## Figures and Tables

**Table 1:** Comparison of the best recognition rates for all tested algorithms.

Algorithms	Recognition rate	Algorithms	Recognition rate
NMF	79.19%	NMFOS	89.06%
DNMF	92.06%	FisherNMF	92.06%
DNTF	95.24%	GDONTF	97.07%
